# The relationship between urinary selenium levels and risk of gestational diabetes mellitus: A nested case–control study

**DOI:** 10.3389/fpubh.2023.1145113

**Published:** 2023-03-27

**Authors:** Yuanxia Liu, Hongmei Chen, Mengtian Zhang, Gangjiao Zhu, Yan Yang, Yuanyuan Li, Wei Lu, Hongling Zhang

**Affiliations:** ^1^College of Medicine and Health, Wuhan Polytechnic University, Wuhan, China; ^2^School of Health and Nursing, Wuchang University of Technology, Wuhan, China; ^3^Key Laboratory of Environment and Health (HUST), Ministry of Education and Ministry of Environmental Protection, State Key Laboratory of Environmental Health, School of Public Health, Tongji Medical College, Huazhong University of Science and Technology, Wuhan, China

**Keywords:** urinary selenium, gestational diabetes mellitus (GDM), nested case–control study, maternal age, fetal sex

## Abstract

**Background:**

Selenium (Se) is an essential trace element for the human body. Serum Se and urinary Se are also biomarkers to assess Se exposure status. However, studies focusing on the association between urinary Se and the risk of gestational diabetes mellitus (GDM) are rare.

**Objective:**

To investigate the association between urinary Se and the risk of GDM.

**Methods:**

A nested case–control study based on a prospective birth cohort in Wuhan, China, which focuses on the effects of prenatal environmental factors exposure on pregnant women and children’s health was conducted. Two hundred and twenty-six cases and 452 controls were included. Maternal urine samples were collected before GDM diagnosis, and the urinary Se levels were determined. We assessed the association of urinary Se with GDM by conditional logistic regression with maternal urinary Se level as a categorical variable, and estimated the association between Se and glucose levels by multiple linear regression. The potential modifier roles of maternal age and fetal sex have also been assessed.

**Results:**

Lower urinary level of Se was significantly associated with a higher risk of GDM (OR = 2.35 for the tertile 1, 95% CI:1.36-4.06; adjusted OR = 1.79 for the tertile 2, 95%CI:1.09-2.95; *p* for trend = 0.01). Fetal sex had an interaction with Se in the association with GDM. The association was more pronounced among pregnant women with female fetuses than with male fetuses.

**Discussion:**

Our study suggested a significant negative association between urinary Se and the risk of GDM, and this association may vary depending on the fetal sex.

## 1. Introduction

Selenium (Se) is an essential trace element for humans and animals (1). As a component of the enzyme glutathione peroxidase, Se protects against oxidative stress damage (2) and also has antioxidant (3), anti-cancer, and anti-viral properties (4). The general population mainly consume Se from eating food, such as grains, meat, and dairy products (5, 6). Deficiency for Se for human body can increase the risks of cardiovascular disease, miscarriage, cancer, and other diseases (7). On the other hand, over-exposure to Se can also be harmful to human health (8).

Gestational diabetes mellitus (GDM) is one of the most common medical complications of pregnancy. As of 2019, the global prevalence of GDM ranges from 7.5 to 27% (9). The prevalence of GDM in China was 14.8% (10), but due to the increasing age of pregnant women, rising obesity rate, unhealthy lifestyle, and more rigorous GDM diagnostic guidelines, it is expected that the prevalence rate of GDM will further increase (11). The International Diabetes Federation has reported that 1 in 6 live births is affected by hyperglycemia in pregnancy (12). GDM is associated with both short-term and long-term adverse health outcomes in mothers and children, such as pre-eclampsia, macrosomia, and increased risks of future type 2 diabetes mellitus (T2DM), obesity, and cardiovascular disease (13). Studies have shown that some factors are associated with the increased risk of GDM, such as age, obesity, metabolic dysregulation, and daily diet (14–17). As an essential trace element, the relationship between Se and the risk of GDM was found. However, their findings are inconsistent. Most recently, a meta-analysis, based on 12 studies, demonstrated that women with GDM had lower Se levels than women without GDM (18). Nevertheless, the difference reported by this meta-analysis was non-significant after correction of the reporting bias by means of the trim-and-fill method (18).

The nested case–control study design can reduce selection bias because both case and control subjects are sampled from the same population, and can also reduce cost and minimize effort because only a fraction of the parent cohort is included (19). Therefore, in the present study, we conducted a nested case–control study based on a large birth cohort study to investigate the relationship between the urinary concentrations of Se of pregnant women and the risk of GDM.

## 2. Materials and methods

### 2.1. The design and population of the study

The design of the present study is a nested case–control study. All GDM cases and controls are from a perspective birth cohort in Wuhan, China, which focuses on the effects of prenatal environmental factors exposure on pregnant women and children’s health. The details of this cohort study have been reported previously (20).

At 24–28 weeks of gestation, pregnant women were given an oral glucose tolerance test (OGTT). Pregnant women need to collect fasting venous blood after 10–12 h of fasting overnight, and then dissolve 75 g of anhydrous glucose in 250-300 ml of water. After drinking for 3–5 min, venous blood will be collected 1 and 2 h after taking sugar. The blood glucose measurement sample shall be venous plasma or serum, and the blood glucose measurement method shall be the glucose oxidase method. Obstetricians of the study hospital would follow the diagnostic criteria of the International Association of Diabetes and Pregnancy Study Groups (IADPSG) (21) that if any of the 75 g OGTT glucose levels met or exceeded the following criteria: fasting: 92 mg/dl (5.1 mmol/l); 1 h after a meal: 180 mg/dl (10.0 mmol/l); and 2 h after meal:153 mg/dl (8.5 mmol/l). The controls were pregnant women who are negative in the OGTT result.

**Figure 1:**
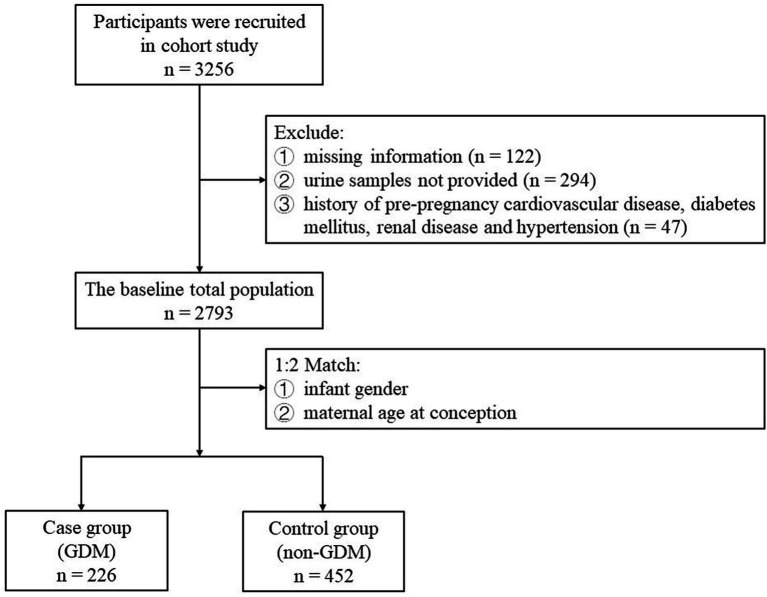
Flowchart of participant recruitment and case–control selection.

The inclusion criteria for our study are: (1) singleton; (2) live births; (3) voluntary participation in this study. The exclusion criteria for our study are: (1) missing information or urine samples not provided; (2) history of pre-pregnancy cardiovascular disease, diabetes mellitus, renal disease and hypertension; (3) multiple pregnancies, birth defect, and stillbirth. For each selected GDM case, two consecutive controls are randomly selected in the birth cohort and matched according to the infant gender and maternal age at conception (that is, the case was matched by two controls of the same age). Women with multiple pregnancies, birth defects, stillbirths, and women whose urine samples could not be analyzed was excluded. In the end, a total of 226 cases and 458 controls were included (Figure 1).

### 2.2. Ethics approval

The study protocol was approved by the ethical committee of the Tongji Medical College, Huazhong University of Science and Technology, and Women and Children Medical and Health care Center of Wuhan. After explaining the detailed description of the research process, each participant signed the informed consent document.

### 2.3. Data collection

Professionally trained nurses conducted face-to-face interviews with pregnant women before the OGTT test or after delivery in the hospital. Demographic and socioeconomic characteristics (e.g., maternal age, residence, employment, household income, education, and self-reported height and weight before pregnancy at the hospital), and lifestyle habits during pregnancy (e.g., exercise, smoking, and alcohol consumption) were collected through the interview. Information about maternal disease history, complications, and birth outcomes were retrieved from the hospital’s medical record. Gestational age was estimated based on the date of the last menstrual period of the pregnant woman. The pre-pregnancy body mass index (BMI) was calculated based on the height and the weight of the pregnant woman before getting pregnant.

### 2.4. Urine sample collection and Se measurements

The maternal mid-stream urine sample was collected in the second trimester (within 3 days before the OGTT). All the mid-stream urine samples collected would be labeled and stored separately in polypropylene tubes and stored at −20°C until further analysis. Determination of Se was carried out by professional laboratory personnel, who could not identify the status of the case and control. Urine samples were thawed at room temperature before analysis, and 1 ml of urine from the supernatant was introduced in Kirgen polypropylene conical centrifuge tubes. Then, 3% HNO_3_ was added to the final volume of 5 ml for overnight nitrification. The resulting sample was digested by ultrasound at 40°C for 1 h and then analyzed using inductively coupled plasma mass spectrometry (Agilent 7,700, Agilent Technologies, Santa Clara, CA, United States). The standard Reference Material Human Urine (SRM2670a, National Institute of Standards and Technology, Gaithersburg, MD, United States) was used as an external quality control, and sample spike-recoveries were used to confirm analytical recovery, which was 95%. A 3% HNO_3_ blank was processed in each batch of samples to control for possible contamination. The samples were analyzed with an external calibration method, using eight standard concentrations ranging from 0 to 500 μg/L. The limit of detection (LOD) for Se in urine was 0.08 μg/L. Field blanks were also included for quality control and the levels of Se in the field blanks were <LOD. The urinary Se concentrations below the LOD were given a value of one-half the LOD. Urinary creatinine concentrations which were determined by the Sarcosine Oxidase Method with Mindray BS-200 CREA Kit (Shenzhen Mindray Bio-medical Electronics Co., Ltd., Shenzhen, China), were used to correct for the effect of variation in urine dilution. Finally, the concentrations of urinary Se were reported as μg/g creatinine.

### 2.5. Statistical analyses

We used the Chi-square test to compare the differences of basic characteristics between GDM cases and controls. After Kolmogorov–Smirnov normality test, we found that Se concentrations in urine samples were skewed distribution. Therefore, we chose to use Wilcoxon rank-sum test to evaluate the difference in the distribution of urinary Se concentrations between cases and controls. Conditional logistic regression models were used to assess the association between maternal urinary Se concentration and the risk of GDM. In the conditional logistic regression models, we used maternal urinary Se levels as a categorical variable which were categorized into three levels. And the criteria for variable conversion is based on the tertile distribution [tertile 1/tertile 2/tertile 3 (T1/T2/T3)] of urinary Se concentrations in the control group. The T3 was assigned as the referent group. The median value with each tertile was used as the score variable in the regression model to test for a linear trend in the risk between GDM and urinary Se concentration.

Maternal education (less than high school, high school, more than high school), pre-pregnancy BMI (<18.5, 18.5–23.9, ≥24 kg/m^2^), and gestational weight gain (<15, 15–20, ≥20 kg) were included in the adjusted models. Because gravid (1, ≥2) has a great impact on GDM, we also include it in the adjustment model (22–24). The association between confounders and GDM were analyzed. Besides, to test the robustness of the results, we conducted the sensitivity analyses by adjusting multivitamin supplement use during pregnancy, hypertension during pregnancy, occupation, and household income, sequentially. The risk estimates were further stratified by maternal age (<30, ≥30 years old) and infant gender. Statistical significance was defined as a two-sided *p* value <0.05. The statistical analyses were performed using the SAS (version 9.4; SAS Institute Inc., Cary, NC, United States).

## 3. Results

The basic characteristics of the cases and controls are shown in Table 1. Among the 678 participants, the mean age of all mothers at delivery was 30.65 ± 4.07 (mean ± SD) years old. Compared with controls, the cases were more likely to be overweight (≥24.0 kg/m^2^), and with lower educational level. The average gestational weight gain (kg) of women in the cases and controls were 14.47 ± 4.81 and 16.12 ± 4.87 (mean ± SD), respectively. The urinary Se levels of pregnant women in GDM cases were significantly lower than those of controls (median: 19.13 vs. 22.24 μg/g creatinine; *p* < 0.05).

**Table 1:** Basic characteristics of GDM cases and controls.

Characteristics	Cases (*n* = 226)	Controls (*n* = 452)	*p*
	*n* (%)	*n* (%)	
Maternal age (years)			1.00
<25	8 (3.54)	16 (3.54)	
25–29	100 (44.25)	200 (44.25)	
30–34	73 (32.30)	146 (32.30)	
≥35	45 (19.91)	90 (19.91)	
Infant sex			1.00
Male	131 (57.96)	262 (57.96)	
Female	95 (42.04)	190 (42.04)	
Pre-pregnancy BMI (kg/m^2^)			0.01
<18.5	20 (8.85)	64 (14.16)	
18.5–23.9	140 (61.95)	324 (71.68)	
≥24.0	66 (29.20)	64 (14.16)	
Education			0.01
Less than high school	12 (5.31)	21 (4.65)	
High school	46 (20.35)	15 (11.28)	
More than high school	168 (74.34)	380 (84.07)	
Household income (yuan per year)			0.60
<50,000	23 (10.18)	55 (12.17)	
≥50,000	202 (89.38)	393 (86.95)	
Missing	1 (0.44)	4 (0.88)	
Gravid			0.06
1	95 (42.04)	225 (49.78)	
≥2	131 (57.96)	227 (50.22)	
Pregnancy-induced hypertension			0.49
No	219 (96.90)	442 (97.79)	
Yes	7 (3.10)	10 (2.21)	
Occupation			0.60
Employed	194 (85.84)	392 (86.73)	
Unemployed	28 (12.39)	56 (12.39)	
Missing	4 (1.77)	4 (0.88)	
Gestational weight gain (kg)			0.01
<15	122 (53.98)	167 (36.95)	
15–20	70 (30.97)	190 (42.04)	
≥20	34 (15.04)	95 (21.02)	
Multivitamin supplement during pregnancy			0.26
No	32 (14.16)	79 (17.52)	
Yes	194 (85.84)	372 (82.48)	
Urinary Se (μg/g creatinine)^a^	19.13 (14.00, 24.94)	22.24 (15.97, 29.34)	

GDM, gestational diabetes mellitus; BMI, body mass index. ^a^Values are presented as median (25–75th percentile).

Table 2 presents the association between maternal urinary Se levels and the odds of GDM. In the crude model, there was a significant increase in the risk of GDM with reduction of Se levels [T3: reference; T2: OR = 2.12 (95%CI: 1.32–3.40); T1: OR = 2.91 (95%CI: 1.71–4.95); *p* for trend <0.01]. After adjustment for potential confounding factors, the dose–response relationship between Se and GDM was still statistically significant [adjusted ORs = 1.79 (95%CI: 1.09–2.95) for the T2 and 2.35 (95%CI: 1.36–4.06) for the T1; *p* for trend <0.01]. The association between each confounder and GDM was provided in Supplementary Table S1. In addition, we conducted sensitivity analysis by adjusting the intake of multiple vitamin supplements during pregnancy, hypertension during pregnancy, occupation, and family income. There is no material change in the observed correlation (Supplementary Table S2).

**Table 2:** Association between maternal urinary Se levels and GDM (*n* = 678).

Se (μg/g creatinine)	Cases	Controls	OR^a^ (95% CI)	OR^b^ (95% CI)
Tertile 1 (<18.10)	96	150	2.91 (1.71–4.95)	2.35 (1.36–4.06)
Tertile 2 (18.10–26.77)	82	151	2.12 (1.32–3.40)	1.79 (1.09–2.95)
Tertile 3 (≥26.77)	48	151	1.00	1.00
*p* for trend			0.01	0.01

OR, odds ratio; CI, confidential interval. ^a^Crude model. ^b^Adjusted for education, gravid, pre-pregnancy BMI, and gestational weight gain.

We further performed analyses stratified by pregnant women’s age and fetal sex (Table 3). In women with age ≥ 30 years old, lower Se concentration was significantly associated with the risk of GDM (T2 of adjusted OR = 1.84, 95%CI: 0.93–3.63; T1 of adjusted OR = 3.31, 95%CI: 1.52–7.23; *p* trend <0.01), but no significant interaction was found (*p* for heterogeneity = 0.14). As for fetal sex, the observed association was more pronounced in pregnant women with female fetuses (adjusted OR = 3.58, 95%CI: 1.30–9.58 for the T1 vs. T3) than those women with male fetuses (adjusted OR = 2.25, 95%CI: 1.16–4.38; *p* for heterogeneity = 0.03).

**Table 3:** The association between maternal urinary Se levels and gestational diabetes mellitus stratified by maternal age and fetal sex.

Se (μg/g creatinine)	Cases/controls (*n*)	OR^a^ (95% CI)	*p* for heterogeneity
Maternal age (years)			**0.14**
**<30 (*n* = 324)**			
Tertile 1 (<17.26)	35/72	1.36(0.60–3.07)	
Tertile 2 (17.26–27.38)	49/72	2.13(1.00–4.55)	
Tertile 3 (≥27.38)	24/72	1.00	
*p* for trend		0.43	
**≥30 (*n* = 354)**			
Tertile 1 (<18.51)	57/79	3.31(1.52–7.23)	
Tertile 2 (18.51–26.48)	38/79	1.84(0.93–3.63)	
Tertile 3 (≥26.48)	23/78	1.00	
*p* for trend		0.01	
Fetal sex			**0.03**
**Male (*n* = 393)**			
Tertile 1 (<19.08)	68/87	2.25 (1.16–4.38)	
Tertile 2 (19.08–27.10)	34/88	1.17 (0.63–2.19)	
Tertile 3 (≥27.10)	29/87	1.00	
*p* for trend		0.01	
**Female (*n* = 285)**			
Tertile 1 (<17.01)	35/63	3.58 (1.30–9.85)	
Tertile 2 (17.01–26.42)	45/64	4.31 (1.68–11.05)	
Tertile 3 (≥26.42)	15/63	1.00	
*p* for trend		0.03	

OR, odds ratio; CI, confidence interval. ^a^Adjusted for education, gravid, pre-pregnancy BMI, gestational weight gain. The bold values “<30 (*n* = 324), ≥30(*n* = 354), Male(*n* = 393), Female (*n* = 285)” meant the stratification by maternal age and fetal sex. The bold values “0.14” and “0.03” meant *p* for heterogeneity.

## 4. Discussion

In this nested case–control study, we found a significant association between decreased urinary Se concentration with the risk of GDM in pregnant women, and this association did not change after adjustment for a series of potential confounding factors. We further found that the association between lower urinary Se and risk of GDM was more pronounced in pregnant women who had female fetuses.

Previous studies have shown that Se in urine is a useful biomarker to assess Se exposure status (25, 26). A comparison of urine Se levels between pregnant women in this study and previously published data is presented in Table 4. Urinary Se levels of our study population (median: 11.9 μg/L and 21.12 μg/g creatinine; geometrical mean (GM): 11.38 μg/L and 20.71 μg/g creatinine) were comparable to those adults in the United Kingdom (median: 13.4 μg/L) (27), but were lower than the general population in Belgium (median: 25.1 μg/L and 21.6 μg/g creatinine) (28). Besides, our study population also had lower levels of urinary Se compared with pregnant women in developed countries, such as Australia (median: 19.1 μg/L and 25.6 μg/g creatinine) (29), Japan (GM: 37.6 μg/g creatinine) (30), the United States (GM: 35.4 μg/g creatinine) (31) and Canada (GM: 44 μg/L) (32). Compare with median urine Se of other countries in our study, the reference significance may be unclear. The possible reason was that considering the heterogeneity of the population, the distribution of urine Se in different populations was also different.

**Table 4:** Comparison of Se levels in urine between the present study and previous studies.

References	Location	Population	*N*	Median	Geometric mean
Present study 2014	Hubei, China	Pregnant women	678	11.91 μg/L	11.38 μg/L
Morton et al. (27)	UK	Adult	1,001	21.12 μg/g creatinine	20.71 μg/g creatinine
Hoet et al. (28)	Belgium	Adult	132	25.1 μg/L	—
Callan et al. (29)	Australia	Pregnant women	173	19.1 μg/L 25.6 μg/g creatinine	—
Shirai et al. (30)	Japan	Pregnant women	78	—	37.6 μg/g creatinine
Kim et al. (31)	USA	Pregnant women	380	—	35.4 μg/g creatinine
Hu et al. (32)	Canada	Women	156	—	44 μg/g creatinine

The association between Se levels during pregnancy and GDM has been investigated in previous studies, but the findings were conflicting. Tan et al. (33) found that the serum Se levels of pregnant women with impaired glucose tolerance and GDM were significantly lower than those of normal pregnant women in a population from Shanghai, China. In a cross-sectional study in Turkey, Kilnc et al. (34) also reported that pregnant women with GDM and those with glucose intolerants had lower Se level than that of the normal pregnant women. Most recently, a meta-analysis, including 12 studies (940 pregnant women with GDM and 1749 controls), suggested that Se levels of women with GDM were lower than those of women without GDM (18). Similar to these above results, our study also found that the urine Se levels of pregnant women in GDM cases were significantly lower than those of controls, and observed a negative correlation between maternal urinary Se concentrations and the risk of GDM. However, there were some controversies about the relationship between Se and GDM in previous studies. Molnar et al. (35) conducted a cross-sectional study in Hungary and reported that the serum Se concentrations of GDM pregnant women were significantly higher than those of normal pregnant women. Liu et al. (36) found that Se levels in the first trimester were not related to GDM in a cohort study. One possible explanation is that this study collected blood samples in the first trimester of pregnancy, while other studies chose the second or third trimester. The Se levels may change in the second or third trimester of pregnancy (37).

We found that the association between the urinary Se levels and the risk of GDM is different in age. Women who were above 30 years old had relatively low urinary Se levels. Similar to our findings, in a cohort study of 506 adults in Attica Province, Greece, Letsiou et al. (38) found that in people aged 18–75, serum Se levels decreased with age. This may be related to the distribution and retention of Se in different tissues of the human body (38). In addition, animal experiments have shown that preserving Se in young rats was more effective than in adult rats (39). Moreover, we found that there was an interaction in the association between maternal urinary Se level and GDM risk by infant sex. There is no reasonable explanation to this finding in existed studies, and further research is needed.

We have discovered several possible mechanisms to explain the link between low Se levels and increased risk of GDM. Firstly, Se has the characteristics of insulin simulation (40), which can promote glucose transport, regulate cell glucose utilization, and ions, and reduce insulin resistance (41). Secondly, a possible mechanism is that Se improves the defense function of the antioxidant system, which may protect β cells to some extent and promote the increase of insulin secretion (42). Thirdly, Se participates in the production of the catalytic site of Se GSHp, which is an enzyme in the body (42). And the activity of enzyme Se GSHp is related to the activation of the nuclear factor-κB (NF-κB) (43). Researchers have proved that the activation of NF-κB is associated with macrovascular complications in late diabetic (44). However, by the role of Se in lipid peroxidation, Se could help to reduce the activity of NF-kB (45).

Nevertheless, there are some limitations in our study. First, the urine Se level of pregnant women was measured only at a certain time point, which may not accurately reflect the Se level of pregnant women during the whole pregnancy. Second, for the dietary information and dyslipidemia of pregnant women, our questionnaire was not comprehensive enough to exclude the possibility of residual confounding. Third, our study was aimed at the Han population in China. In the future, the daily dietary intake questionnaire should be added to comprehensively analyze the relationship between Se levels and GDM. Future researchers can conduct prospective cohort studies to investigate urinary Se status in populations of different ethnicities and countries.

In this nested case–control study, we found that a correlation between low urinary Se concentration in pregnant women and increased risk of GDM in pregnant women. This association suggests that low Se concentration during pregnancy may be one of the risk factors for GDM. In the future, more in-depth studies are needed to find out the possible mechanisms, and to provide the basis for guiding pregnant women to supplement Se reasonably.

## Data availability statement

The raw data supporting the conclusions of this article will be made available by the authors, without undue reservation.

## Ethics statement

The study was conducted according to the guidelines of the Declaration of Helsinki, and approved by the ethics committee of Tongji Medical College, Huazhong University of Science and Technology (no. [2014] 14#), and the study hospital (no. 2010009). The patients/participants provided their written informed consent to participate in this study.

## Author contributions

YLiu: conceptualization, methodology, software, formal analysis, and writing—original draft preparation. HC: investigation and validation. MZ: investigation. GZ: data curation. YY: data validation. YLi: project administration and funding acquisition. WL: resources, writing—review and editing, and supervision. HZ: conceptualization, writing—review and editing, and resources. All authors contributed to the article and approved the submitted version.

## Funding

This work was supported by grants from the National Key Research and Development Plan (2022YFE0132900), the Program for HUST Academic Frontier Youth Team (2018QYTD12) and the National Institutes of Health R01ES029082.

## Conflict of interest

The authors declare that the research was conducted in the absence of any commercial or financial relationships that could be construed as a potential conflict of interest.

## Publisher’s note

All claims expressed in this article are solely those of the authors and do not necessarily represent those of their affiliated organizations, or those of the publisher, the editors and the reviewers. Any product that may be evaluated in this article, or claim that may be made by its manufacturer, is not guaranteed or endorsed by the publisher.

## Supplementary material

The Supplementary material for this article can be found online at: https://www.frontiersin.org/articles/10.3389/fpubh.2023.1145113/full#supplementary-material
