# Supplementary material for: The relationship between urinary selenium levels and risk of gestational diabetes mellitus: A nested case–control study
Source: Front Public Health. 2023 Mar 27;11:1145113. doi: 10.3389/fpubh.2023.1145113 (PMC10083259; doi:10.3389/fpubh.2023.1145113)
Supplement: Supplementary file 1 [file Data_Sheet_1.docx]

**Table S1. The association between confounders and GDM.**

| Confounder | OR (*95%CI*) | |
| --- | --- | --- |
| Education | |  |
| Less than high school | | 1 |
| High school | | 1.7 (0.73, 3.95) |
| More than high school | | 0.94 (0.44, 2.01) |
| Gravid | |  |
| 1 | | 1 |
| ≥ 2 | | 1.24 (0.84, 1.83) |
| Pre-pregnancy BMI (kg/m^2^) | |  |
| < 18.5 | | 1 |
| 18.5–23.9 | | 1.25 (0.7, 2.24) |
| ≥ 24.0 | | 2.63 (1.32,5.23) |
| Gestational weight gain (kg) | |  |
| < 15 | | 1 |
| 15-20 | | 0.58 (0.4, 0.83) |
| ≥ 20 | | 0.58 (0.36, 0.95) |

**Table S2**. **The Association between maternal urinary Se levels and GDM after sequentially adjusting covariates.**

|  | OR (95%CI) | |
| --- | --- | --- |
|  | Tertile 2^e^ | Tertile 1 ^f^ |
| multivitamin supplement use during pregnancy ^a^ | \| 1.79 (1.09, 2.96) \| \| --- \| | \| 2.43 (1.40, 4.22) \| \| --- \| |
| hypertension during pregnancy ^b^ | \| 1.79 (1.08, 2.95) \| \| --- \| | \| 2.43 (1.40, 4.23) \| \| --- \| |
| occupation ^c^ | \| 1.77 (1.07, 2.93) \| \| --- \| | \| 2.42 (1.38, 4.24) \| \| --- \| |
| household income ^d^ | \| 1.79 (1.08, 2.97) \| \| --- \| | \| 2.49 (1.42, 4.35) \| \| --- \| |

Abbreviation: OR, odds ratio; CI, confidential interval.

^a^ Adjusted for education, gravid, pre-pregnancy BMI, gestational weight gain and multivitamin supplement use during pregnancy

^b^ Adjusted for education, gravid, pre-pregnancy BMI, gestational weight gain, multivitamin supplement use during pregnancy and hypertension during pregnancy

^c^ Adjusted for education, gravid, pre-pregnancy BMI, gestational weight gain, multivitamin supplement use during pregnancy, hypertension during pregnancy and occupation

^d^ Adjusted for education, gravid, pre-pregnancy BMI, gestational weight gain, multivitamin supplement use during pregnancy, hypertension during pregnancy,occupation and household income

^e^ Tertile 2group compared to the reference group (Tertile 3)

^f^ Tertile 1group compared to the reference group (Tertile 3)
